# Supplementary material for: A Subset of Oligodendrocyte Lineage Cells Interact With the Developing Dorsal Root Entry Zone During Its Genesis
Source: Front Cell Neurosci. 2022 Jun 6;16:893629. doi: 10.3389/fncel.2022.893629 (PMC9207214; doi:10.3389/fncel.2022.893629)
Supplement: Supplementary file 2 [file Data_Sheet_1.pdf]

SUPPLEMENTARY INFORMATION:

Table S1. List of the transgenic zebrafish strains used in the study.

**Transgenic animal**	**Labels**
**AB**	Wildtype animal, no transgenic labeling
Tg(sox10:mrfp)	Spinal cord; oligodendrocytes, neurons, and dorsal root ganglia (DRG)
Tg(sox10:megfp)	Spinal cord; oligodendrocytes, neurons, and dorsal root ganglia (DRG)
Tg(mbpa:egfp)	Oligodendrocytes expressing *mbpa*$^{+}$ myelin basic protein
Tg(sox10:eos)	Photoconvertible line that labels oligodendrocytes and neurons. Unconverted fluoresces green. Converted fluoresces red.
Tg(sox10:gal4;uas:lifeact-gfp)	Actin within oligodendrocytes, neurons, and dorsal root ganglia (DRG)
Tg(nkx2.2a:gfp)	Oligodendrocytes derived from the floor plate region of the spinal cord
Tg(gfap:nsfb-mcherry)	Radial glia
Tg(olig2:dsred)	Oligodendrocytes derived from the pMN domain of the spinal cord
Tg(dbx:gfp)	Oligodendrocytes derived from the dbx domain of the spinal cord

Tg(nbt:dsred)	Pan-neuronal marker
Tg(ngn1:gfp)	Sensory nerves

Table S2. Statistics Sheet. All raw values of the individual cell and zebrafish n-values scored for each figure. Table also includes specific values for statistical tests and exact p-values used to determine statistical significance for each figure panel.

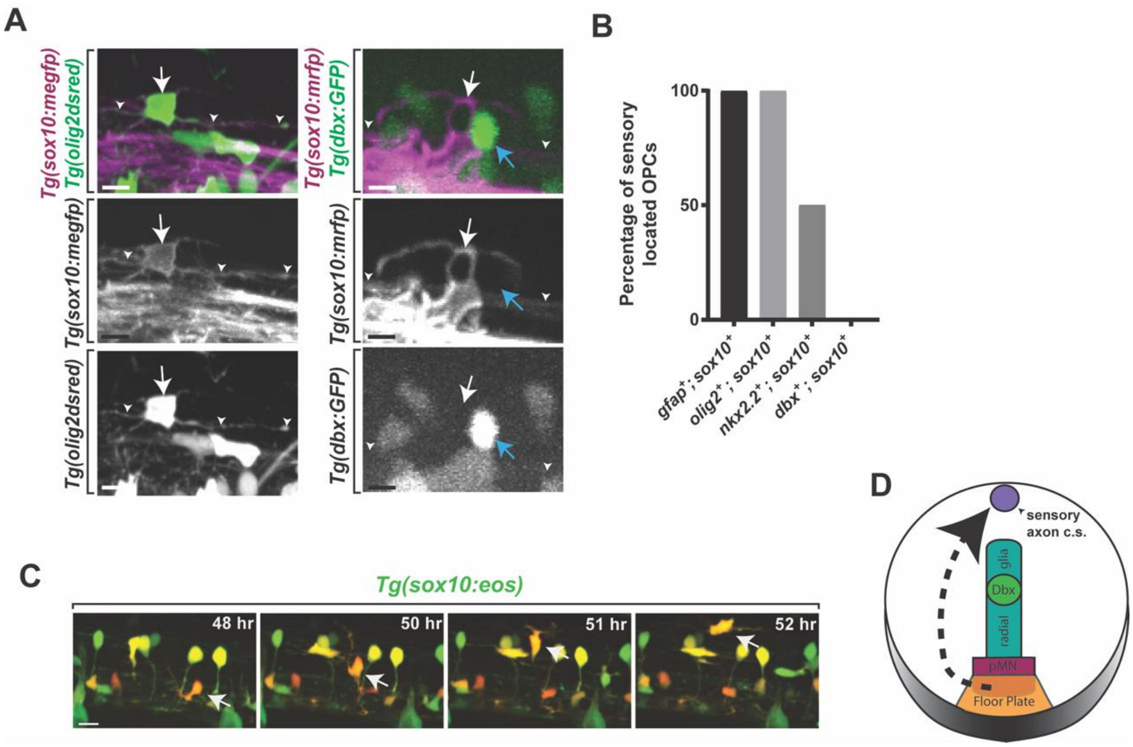

Figure S1. Sensory oligodendrocytes are not generated from a distinct progenitor pool. (A) Confocal z-stack still images taken at 72 hpf in *Tg(sox10:megfp);Tg(olig2:dsred),* and *Tg(sox10:mrfp);Tg(dbx:gfp)* animals showing the presence or absence of progenitor marker expression in sensory OLs. White arrows represent sensory OLs. Blue arrow represents nonsensory OL. White arrowheads represent the sensory axon. (B) Quantification of the percentage of sensory located oligodendrocytes that are *olig2*$^{+}$*;sox10*$^{+}$, *nkx2.2a*$^{+}$*;sox10*$^{+}$*, or dbx*$^{+}$*;sox10.*$^{+}$ (C) Images from a 24 hour time-lapse starting at 48 hpf in *Tg(sox10:eos)* animals showing the migration of a photoconverted OPC from the ventral spinal cord to the dorsal spinal cord region. White arrow represents a photoconverted OPC that migrates to the sensory. (D) Schematic diagram depicting the migration of sensory oligodendrocytes from the floor plate region (*nkx2.2a*) to the sensory axon. Scale bar equals 10µm (A,C). All images are orientated anterior to left, posterior to right, dorsal up and ventral down.

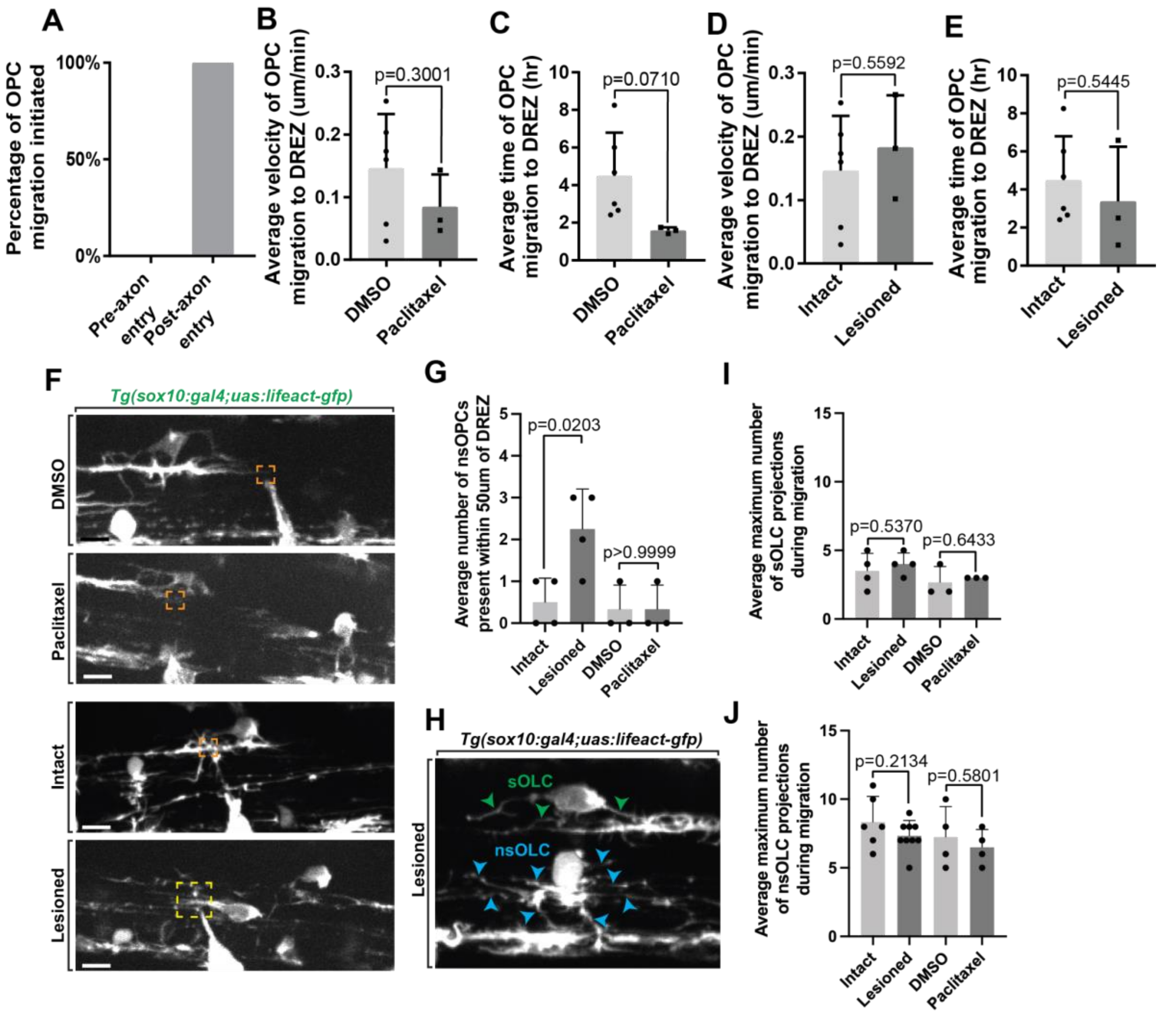

Figure S2. Sensory-related OLCs arrive following axon entry and DREZ contact. (A) Quantification of the percentage of OPC migration initiated showing OPC migration occurs post-axon entry. (B) Quantification of the average velocity of OPC initiation to the DREZ in DMSO and Paclitaxel treated animals (p=0.3001). (C) Quantification of the average time of OPC migration initiation to the DREZ in DMSO and Paclitaxel treated animals (p=0.0710). (D) Quantification of the average velocity of OPC initiation to the DREZ in intact and lesioned treated animals (p=0.5592). (E) Quantification of the average time of OPC migration initiation to the DREZ in intact and lesioned treated animals (p=0.5445). (F) Images from a 24 hour time-lapse movie starting at 48 hpf in *Tg(sox10:gal4 ;*

uas:lifeact-gfp) zebrafish showing nonsensory OPCs responding more to lesioned animals compared to intact animals and DMSO and Paclitaxel treated animals. Yellow dashed box indicates false DREZ created by lesioning the animal. Orange box indicates DREZ formation. (G) Quantification showing average number of nonsensory OPCs present within a 50μm window of the DREZ ($p=0.0203$, $p>0.999$). (H) Image from a 24 hour time-lapse movie of the projections of both sensory and nonsensory OLCs in an individual lesioned *Tg(sox10:gal4 ; uas:lifeact-gfp)* zebrafish. Green arrowheads indicate sensory OLCs. Blue arrowheads indicate nonsensory OLCs. (I) Quantification of the maximum number of projections a sensory OLC created migrating to the DREZ in intact and DMSO treated control compared to lesioned and Paclitaxel treated animals ($p=0.5370$, $p=0.6433$). (J) Same quantification represented in (I) but of the number of projections nonsensory OLCs created during migration to the DREZ ($p=0.2134$, $p=0.5801$). Scale bar equals 10μm (F). All images are orientated anterior to left, posterior to right, dorsal up and ventral down.
